# Chlorido(2-chloro­nicotinato)triphenyl­anti­mony(V)

**DOI:** 10.1107/S1600536808029632

**Published:** 2008-09-20

**Authors:** Li Quan, Handong Yin, Daqi Wang

**Affiliations:** a College of Chemistry and Chemical Engineering, Liaocheng University, Shandong 252059, People’s Republic of China

## Abstract

In the title complex, [Sb(C_6_H_5_)_3_(C_6_H_3_ClNO_2_)Cl], the Sb center has a close to ideal trigonal-bipyramidal geometry, with the phenyl ligands in equatorial positions and the chloride and a carboxyl­ate O atom in axial positions. Weak C—H⋯O contacts generate dimeric units *via* crystallographic inversion centres.

## Related literature

For related structures, see: Yin *et al.* (2008 ▶); Chaudhari *et al.* (2007 ▶)
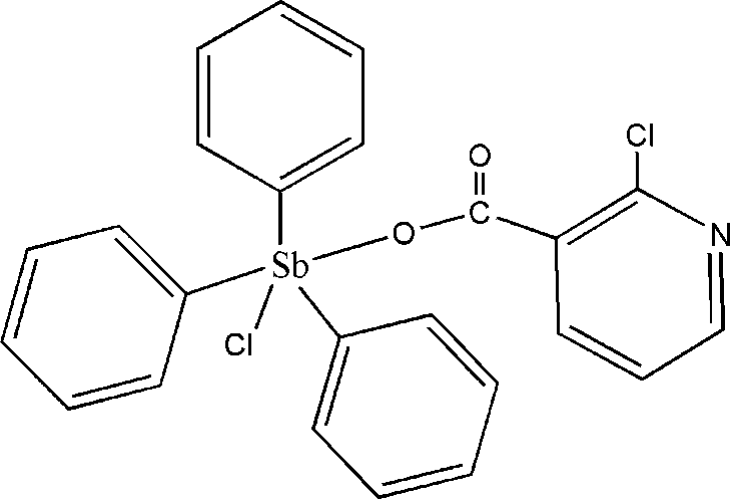


## Experimental

### 

#### Crystal data

[Sb(C_6_H_5_)_3_(C_6_H_3_ClNO_2_)Cl]
*M* *_r_* = 545.04
Monoclinic, 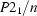

*a* = 11.9580 (9) Å
*b* = 15.4428 (18) Å
*c* = 12.0264 (15) Å
β = 94.291 (2)°
*V* = 2214.6 (4) Å^3^
*Z* = 4
Mo *K*α radiation
μ = 1.51 mm^−1^
*T* = 298 (2) K
0.45 × 0.41 × 0.40 mm

#### Data collection

Bruker SMART CCD diffractometer
Absorption correction: multi-scan (*SADABS*; Sheldrick, 1996 ▶) *T*_min_ = 0.550, *T*_max_ = 0.584 (expected range = 0.516–0.547)
10922 measured reflections
3893 independent reflections
3149 reflections with *I* > 2σ(*I*)
*R*_int_ = 0.026

#### Refinement

*R*[*F* ^2^ > 2σ(*F* ^2^)] = 0.023
*wR*(*F* ^2^) = 0.071
*S* = 1.00
3893 reflections
271 parameters
H-atom parameters constrained
Δρ_max_ = 0.52 e Å^−3^
Δρ_min_ = −0.39 e Å^−3^

### 

Data collection: *SMART* (Siemens, 1996 ▶); cell refinement: *SAINT* (Siemens, 1996 ▶); data reduction: *SAINT*; program(s) used to solve structure: *SHELXS97* (Sheldrick, 2008 ▶); program(s) used to refine structure: *SHELXL97* (Sheldrick, 2008 ▶); molecular graphics: *SHELXTL* (Sheldrick, 2008 ▶); software used to prepare material for publication: *SHELXTL*.

## Supplementary Material

Crystal structure: contains datablocks I, global. DOI: 10.1107/S1600536808029632/si2109sup1.cif

Structure factors: contains datablocks I. DOI: 10.1107/S1600536808029632/si2109Isup2.hkl

Additional supplementary materials:  crystallographic information; 3D view; checkCIF report

## Figures and Tables

**Table:** 

Sb1—C19	2.107 (3)
Sb1—C13	2.111 (3)
Sb1—C7	2.111 (3)
Sb1—O1	2.114 (2)
Sb1—Cl2	2.4921 (8)

**Table:** 

C19—Sb1—C13	137.38 (12)
C19—Sb1—C7	114.05 (12)
C13—Sb1—C7	108.55 (12)
O1—Sb1—Cl2	177.56 (6)

**Table 2:** Hydrogen-bond geometry (Å, °)

*D*—H⋯*A*	*D*—H	H⋯*A*	*D*⋯*A*	*D*—H⋯*A*
C20—H20⋯O2^i^	0.93	2.59	3.346 (4)	139

Symmetry code: (i) 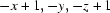.

**Fig. 1.**
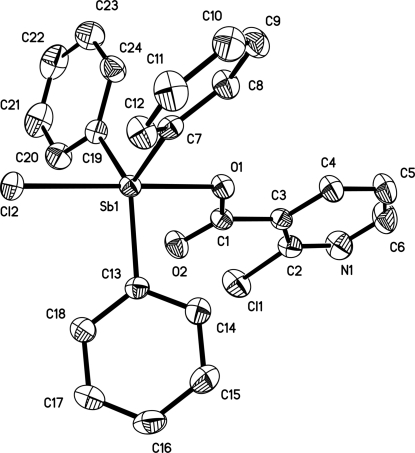
The molecular structure of (I) with atom labels and 50% probability displacement ellipsoids.

**Fig. 2.**
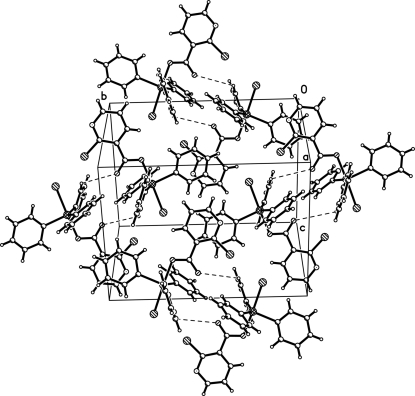
A dimer linked by C20—H20···O2i hydrogen bonds (dashed lines) (symmetry code i: 1 - x,-y,1 - z).

## supplementary crystallographic information

### Comment

The triphenylantimony(V) acetylferroceneoxime structure shows some *in vitro* antitumor activity (Yin *et al.* 2008). The title compound may show similar activities. A similar compound was synthesized (Chaudhari *et al.*2007). The crystal structure of complex I (Fig. 1) consists of dimeric units, (Fig. 2) generated through weak intermolecular C—H···O hydrogen bonds *via* crystallographic inversion centres (Table 2). The sum of the equatorial angles C7—Sb1—C13, C13—Sb1—C19 and C19—Sb1—C7 is 359.98° and the corresponding axial angle Cl2—Sb1—O1 is 177.56 (6)° (Table 1). The distance Sb—O1 2.114 (2) Å is shorter than the range of short Sb—O distances 2.119 (3) - 2.133 (3) Å, and shorter than the range of the long Sb—O distances between 3.012 (6) and 3.112 (4) Å from related literature (Chaudhari *et al.*2007). The Sb1—O2 distance of 2.898 (2) Å in the title complex is shorter than the sum of the van der Waals radii for Sb and O (3.2 Å), but much longer than the covalent bond Sb–O1 (2.114 (2) Å). So it can be considered that atom O2 does not make any significant contact with the Sb1 atom.

### Experimental

2-chloronicotinic acid (0.044 g, 0.4 mmol) and sodium methoxide (0.8 ml, 0.4 mmol) was added to a stirring solution containing triphenylantimonydichloride (0.172 g, 0.4 mmol) in toluene (25 ml). After refluxing for 8 h, the colorless solution was obtained and then filtered. The solvent was gradually removed by evaporation under vacuum until the white solid was obtained. The solid was recrystallized from petroleum ether/dichoromethane (1:1) to give colorless crystals.

### Refinement

All H atoms were placed in calculated positions, with C—H = 0.93 Å, with *U*_iso_(H) = 1.2 *U*_eq_(C).

### Crystal data

**Table:**

[Sb(C_6_H_5_)_3_(C_6_H_3_ClNO_2_)Cl]	*F*(000) = 1080
*M**_r_* = 545.04	*D*_x_ = 1.635 Mg m^−^^3^
Monoclinic, *P*2_1_/*n*	Mo *K*α radiation, λ = 0.71073 Å
Hall symbol: -P 2yn	Cell parameters from 5771 reflections
*a* = 11.9580 (9) Å	θ = 2.3–28.0°
*b* = 15.4428 (18) Å	µ = 1.51 mm^−^^1^
*c* = 12.0264 (15) Å	*T* = 298 K
β = 94.291 (2)°	Block, colorless
*V* = 2214.6 (4) Å^3^	0.45 × 0.41 × 0.40 mm
*Z* = 4	

### Data collection

**Table:**

Bruker SMART CCD diffractometer	3893 independent reflections
Radiation source: fine-focus sealed tube	3149 reflections with *I* > 2σ(*I*)
graphite	*R*_int_ = 0.026
φ and ω scans	θ_max_ = 25.0°, θ_min_ = 2.2°
Absorption correction: multi-scan (SADABS; Sheldrick, 1996)	*h* = −13→14
*T*_min_ = 0.550, *T*_max_ = 0.584	*k* = −17→18
10922 measured reflections	*l* = −14→10

### Refinement

**Table:**

Refinement on f^2^	Primary atom site location: structure-invariant direct methods
Least-squares matrix: full	Secondary atom site location: difference Fourier map
*R*[*F*^2^ > 2σ(*F*^2^)] = 0.023	Hydrogen site location: inferred from neighbouring sites
*wR*(*F*^2^) = 0.071	H-atom parameters constrained
*S* = 1.00	*w* = 1/[σ^2^(*F*_o_^2^) + (0.0415*P*)^2^ + 0.4912*P*] where *P* = (*F*_o_^2^ + 2*F*_c_^2^)/3
3893 reflections	(Δ/σ)_max_ = 0.001
271 parameters	Δρ_max_ = 0.52 e Å^−^^3^
0 restraints	Δρ_min_ = −0.39 e Å^−^^3^

### Fractional atomic coordinates and isotropic or equivalent isotropic displacement parameters (Å^2^)

**Table:**

	*x*	*y*	*z*	*U*_iso_*/*U*_eq_	
Sb1	0.523870 (16)	0.223556 (12)	0.600425 (16)	0.03369 (9)	
Cl1	0.55909 (9)	−0.11472 (6)	0.80420 (9)	0.0663 (3)	
Cl2	0.45726 (7)	0.27660 (6)	0.41124 (6)	0.0467 (2)	
N1	0.6489 (3)	−0.0695 (2)	0.9960 (3)	0.0674 (9)	
O1	0.58192 (17)	0.17341 (13)	0.75786 (17)	0.0412 (5)	
O2	0.5294 (2)	0.04741 (15)	0.68384 (19)	0.0534 (6)	
C1	0.5703 (2)	0.0892 (2)	0.7620 (3)	0.0389 (7)	
C2	0.6110 (3)	−0.0368 (2)	0.8986 (3)	0.0472 (8)	
C3	0.6124 (2)	0.0507 (2)	0.8723 (3)	0.0390 (7)	
C4	0.6553 (3)	0.1057 (2)	0.9560 (3)	0.0526 (9)	
H4	0.6581	0.1650	0.9431	0.063*	
C5	0.6938 (3)	0.0727 (3)	1.0584 (3)	0.0671 (11)	
H5	0.7220	0.1092	1.1153	0.081*	
C6	0.6895 (4)	−0.0142 (3)	1.0742 (4)	0.0746 (13)	
H6	0.7163	−0.0363	1.1431	0.090*	
C7	0.5622 (3)	0.34849 (19)	0.6637 (3)	0.0382 (7)	
C8	0.6477 (3)	0.3615 (2)	0.7465 (3)	0.0498 (9)	
H8	0.6901	0.3151	0.7753	0.060*	
C9	0.6690 (3)	0.4453 (2)	0.7860 (3)	0.0584 (10)	
H9	0.7269	0.4549	0.8406	0.070*	
C10	0.6060 (4)	0.5124 (2)	0.7453 (3)	0.0629 (11)	
H10	0.6214	0.5680	0.7719	0.075*	
C11	0.5199 (4)	0.4995 (2)	0.6655 (3)	0.0657 (11)	
H11	0.4758	0.5459	0.6395	0.079*	
C12	0.4988 (3)	0.4171 (2)	0.6237 (3)	0.0537 (9)	
H12	0.4414	0.4084	0.5683	0.064*	
C13	0.3560 (2)	0.1945 (2)	0.6294 (3)	0.0363 (7)	
C14	0.3242 (3)	0.2102 (2)	0.7346 (3)	0.0527 (9)	
H14	0.3771	0.2264	0.7914	0.063*	
C15	0.2124 (3)	0.2015 (3)	0.7553 (3)	0.0649 (11)	
H15	0.1895	0.2136	0.8259	0.078*	
C16	0.1358 (3)	0.1752 (3)	0.6727 (3)	0.0584 (10)	
H16	0.0609	0.1693	0.6872	0.070*	
C17	0.1683 (3)	0.1575 (3)	0.5685 (3)	0.0612 (10)	
H17	0.1158	0.1383	0.5131	0.073*	
C18	0.2789 (3)	0.1682 (2)	0.5453 (3)	0.0510 (9)	
H18	0.3010	0.1578	0.4740	0.061*	
C19	0.6570 (3)	0.1635 (2)	0.5247 (2)	0.0388 (7)	
C20	0.6410 (3)	0.0945 (2)	0.4541 (3)	0.0559 (9)	
H20	0.5701	0.0701	0.4409	0.067*	
C21	0.7306 (4)	0.0614 (3)	0.4028 (3)	0.0727 (12)	
H21	0.7202	0.0144	0.3548	0.087*	
C22	0.8339 (4)	0.0967 (3)	0.4217 (4)	0.0769 (14)	
H22	0.8942	0.0735	0.3874	0.092*	
C23	0.8497 (3)	0.1667 (3)	0.4913 (4)	0.0723 (13)	
H23	0.9206	0.1913	0.5026	0.087*	
C24	0.7621 (3)	0.2011 (3)	0.5444 (3)	0.0544 (9)	
H24	0.7729	0.2482	0.5922	0.065*	

### Atomic displacement parameters (Å^2^)

**Table:**

	*U*^11^	*U*^22^	*U*^33^	*U*^12^	*U*^13^	*U*^23^
Sb1	0.03589 (13)	0.02844 (13)	0.03649 (13)	−0.00131 (9)	0.00101 (9)	0.00020 (9)
Cl1	0.0809 (7)	0.0349 (5)	0.0805 (7)	−0.0049 (5)	−0.0123 (5)	0.0016 (5)
Cl2	0.0547 (5)	0.0472 (5)	0.0381 (4)	0.0028 (4)	0.0029 (4)	0.0067 (4)
N1	0.078 (2)	0.055 (2)	0.067 (2)	0.0044 (18)	−0.0095 (18)	0.0224 (18)
O1	0.0499 (12)	0.0300 (12)	0.0424 (12)	−0.0017 (10)	−0.0044 (10)	0.0024 (10)
O2	0.0722 (16)	0.0367 (13)	0.0488 (14)	−0.0085 (12)	−0.0122 (12)	0.0001 (11)
C1	0.0393 (17)	0.0341 (18)	0.0429 (18)	−0.0006 (14)	0.0007 (14)	−0.0014 (15)
C2	0.0437 (18)	0.043 (2)	0.055 (2)	0.0016 (16)	−0.0005 (16)	0.0041 (17)
C3	0.0362 (16)	0.0378 (18)	0.0423 (18)	0.0015 (14)	−0.0006 (13)	0.0010 (14)
C4	0.061 (2)	0.045 (2)	0.050 (2)	−0.0025 (17)	−0.0085 (17)	0.0012 (17)
C5	0.077 (3)	0.071 (3)	0.049 (2)	−0.002 (2)	−0.017 (2)	−0.001 (2)
C6	0.091 (3)	0.077 (3)	0.052 (2)	0.005 (3)	−0.018 (2)	0.017 (2)
C7	0.0446 (17)	0.0273 (16)	0.0438 (18)	−0.0015 (14)	0.0103 (15)	−0.0004 (14)
C8	0.061 (2)	0.037 (2)	0.051 (2)	−0.0001 (17)	−0.0046 (17)	−0.0006 (16)
C9	0.069 (2)	0.051 (2)	0.054 (2)	−0.012 (2)	−0.0057 (19)	−0.0107 (18)
C10	0.093 (3)	0.033 (2)	0.063 (3)	−0.009 (2)	0.007 (2)	−0.0060 (18)
C11	0.092 (3)	0.036 (2)	0.068 (3)	0.012 (2)	−0.005 (2)	−0.0024 (19)
C12	0.063 (2)	0.038 (2)	0.058 (2)	0.0060 (17)	−0.0044 (18)	−0.0056 (17)
C13	0.0349 (16)	0.0318 (17)	0.0420 (18)	0.0006 (13)	0.0026 (14)	0.0061 (13)
C14	0.048 (2)	0.066 (3)	0.045 (2)	−0.0009 (18)	0.0041 (16)	−0.0008 (17)
C15	0.055 (2)	0.087 (3)	0.055 (2)	0.000 (2)	0.0178 (19)	0.001 (2)
C16	0.0376 (18)	0.065 (3)	0.074 (3)	0.0008 (18)	0.0122 (19)	0.013 (2)
C17	0.0403 (19)	0.076 (3)	0.067 (3)	−0.0089 (19)	−0.0027 (18)	−0.002 (2)
C18	0.0430 (19)	0.062 (2)	0.048 (2)	−0.0040 (18)	0.0016 (16)	−0.0052 (18)
C19	0.0426 (17)	0.0341 (17)	0.0398 (17)	0.0059 (14)	0.0038 (14)	0.0010 (14)
C20	0.069 (2)	0.041 (2)	0.058 (2)	−0.0052 (18)	0.0049 (19)	−0.0097 (17)
C21	0.101 (3)	0.054 (3)	0.065 (3)	0.023 (3)	0.024 (2)	−0.009 (2)
C22	0.075 (3)	0.090 (4)	0.068 (3)	0.042 (3)	0.024 (2)	0.011 (3)
C23	0.041 (2)	0.101 (4)	0.075 (3)	0.012 (2)	0.004 (2)	0.006 (3)
C24	0.0442 (19)	0.064 (2)	0.055 (2)	0.0044 (18)	−0.0025 (17)	−0.0053 (18)

### Geometric parameters (Å, °)

**Table:**

Sb1—C19	2.107 (3)	C11—C12	1.385 (5)
Sb1—C13	2.111 (3)	C11—H11	0.9300
Sb1—C7	2.111 (3)	C12—H12	0.9300
Sb1—O1	2.114 (2)	C13—C14	1.369 (5)
Sb1—Cl2	2.4921 (8)	C13—C18	1.377 (4)
Cl1—C2	1.736 (4)	C14—C15	1.385 (5)
N1—C2	1.324 (4)	C14—H14	0.9300
N1—C6	1.335 (5)	C15—C16	1.361 (5)
O1—C1	1.309 (4)	C15—H15	0.9300
O2—C1	1.213 (4)	C16—C17	1.367 (5)
C1—C3	1.506 (4)	C16—H16	0.9300
C2—C3	1.389 (5)	C17—C18	1.382 (5)
C3—C4	1.386 (5)	C17—H17	0.9300
C4—C5	1.380 (5)	C18—H18	0.9300
C4—H4	0.9300	C19—C20	1.367 (5)
C5—C6	1.356 (6)	C19—C24	1.390 (5)
C5—H5	0.9300	C20—C21	1.374 (5)
C6—H6	0.9300	C20—H20	0.9300
C7—C12	1.369 (5)	C21—C22	1.354 (6)
C7—C8	1.388 (4)	C21—H21	0.9300
C8—C9	1.394 (5)	C22—C23	1.371 (6)
C8—H8	0.9300	C22—H22	0.9300
C9—C10	1.351 (5)	C23—C24	1.373 (5)
C9—H9	0.9300	C23—H23	0.9300
C10—C11	1.369 (5)	C24—H24	0.9300
C10—H10	0.9300		
			
C19—Sb1—C13	137.38 (12)	C10—C11—C12	119.7 (4)
C19—Sb1—C7	114.05 (12)	C10—C11—H11	120.1
C13—Sb1—C7	108.55 (12)	C12—C11—H11	120.1
C19—Sb1—O1	91.02 (10)	C7—C12—C11	120.2 (3)
C13—Sb1—O1	91.53 (10)	C7—C12—H12	119.9
C7—Sb1—O1	87.95 (10)	C11—C12—H12	119.9
C19—Sb1—Cl2	87.14 (8)	C14—C13—C18	120.9 (3)
C13—Sb1—Cl2	88.72 (8)	C14—C13—Sb1	116.6 (2)
C7—Sb1—Cl2	94.27 (9)	C18—C13—Sb1	122.3 (2)
O1—Sb1—Cl2	177.56 (6)	C13—C14—C15	119.2 (4)
C2—N1—C6	117.5 (3)	C13—C14—H14	120.4
C1—O1—Sb1	111.67 (19)	C15—C14—H14	120.4
O2—C1—O1	122.5 (3)	C16—C15—C14	120.2 (4)
O2—C1—C3	124.1 (3)	C16—C15—H15	119.9
O1—C1—C3	113.4 (3)	C14—C15—H15	119.9
N1—C2—C3	124.3 (3)	C15—C16—C17	120.5 (3)
N1—C2—Cl1	113.3 (3)	C15—C16—H16	119.8
C3—C2—Cl1	122.4 (3)	C17—C16—H16	119.8
C4—C3—C2	116.2 (3)	C16—C17—C18	120.2 (3)
C4—C3—C1	118.6 (3)	C16—C17—H17	119.9
C2—C3—C1	125.2 (3)	C18—C17—H17	119.9
C5—C4—C3	120.1 (4)	C13—C18—C17	119.0 (3)
C5—C4—H4	119.9	C13—C18—H18	120.5
C3—C4—H4	119.9	C17—C18—H18	120.5
C6—C5—C4	118.5 (4)	C20—C19—C24	121.0 (3)
C6—C5—H5	120.7	C20—C19—Sb1	122.5 (3)
C4—C5—H5	120.7	C24—C19—Sb1	116.4 (2)
N1—C6—C5	123.3 (4)	C19—C20—C21	119.4 (4)
N1—C6—H6	118.3	C19—C20—H20	120.3
C5—C6—H6	118.3	C21—C20—H20	120.3
C12—C7—C8	119.9 (3)	C22—C21—C20	120.5 (4)
C12—C7—Sb1	118.8 (2)	C22—C21—H21	119.8
C8—C7—Sb1	121.3 (2)	C20—C21—H21	119.8
C7—C8—C9	119.0 (3)	C21—C22—C23	120.2 (4)
C7—C8—H8	120.5	C21—C22—H22	119.9
C9—C8—H8	120.5	C23—C22—H22	119.9
C10—C9—C8	120.4 (4)	C22—C23—C24	120.9 (4)
C10—C9—H9	119.8	C22—C23—H23	119.5
C8—C9—H9	119.8	C24—C23—H23	119.5
C9—C10—C11	120.8 (4)	C23—C24—C19	118.0 (4)
C9—C10—H10	119.6	C23—C24—H24	121.0
C11—C10—H10	119.6	C19—C24—H24	121.0

### Hydrogen-bond geometry (Å, °)

**Table:**

*D*—H···*A*	*D*—H	H···*A*	*D*···*A*	*D*—H···*A*
C20—H20···O2^i^	0.93	2.59	3.346 (4)	139.

Symmetry codes: (i) −*x*+1, −*y*, −*z*+1.
